# Defining paediatric neurorehabilitation: You cannot improve what you cannot characterize

**DOI:** 10.1111/dmcn.15919

**Published:** 2024-04-26

**Authors:** Rob Forsyth, John Whyte

**Affiliations:** ^1^ Translational and Clinical Research Institute Newcastle University Newcastle upon Tyne UK; ^2^ Moss Rehabilitation Research Institute Elkins Park PA USA

## Abstract

Neurorehabilitation is the primary therapy for neurological impairment in children, yet its potential to achieve change remains incompletely understood and probably underestimated. Understanding ‘the difference neurorehabilitation can make’ against a background of neurological repair and recovery as well as ongoing neurological development is an enormous challenge, exacerbated to no small extent by the lack of a ‘common currency’ for the description and measurement of the neurorehabilitation services a child is receiving. This review addresses attempts to parse neurorehabilitation treatment content in theoretically and mechanistically valid ways that might help address this challenge.

## Graphical abstract

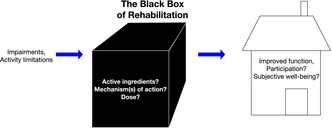

This invited review is commented by Carroll on pages 1115–1116 of this issue.

**Abbreviations**
PRISM: Paediatric Rehabilitation IngredientS Measure
RTSS: Rehabilitation Treatment Specification System

What this paper adds

- Identifying the effects of neurorehabilitation treatment is challenging.
- Part of the challenge is robust identification of active ingredients and mechanisms of action.
- There should be clear hypotheses about the mechanisms of action of rehabilitation ingredients.
- This will allow ingredients' efficacy to be evaluated against those aspects of outcome predicted to change.

Rehabilitation comprises a set of clinical services intended to enhance functioning and quality of life after illness or injury. Neurorehabilitation—rehabilitation applied to neurological conditions—is a mainstay of the clinical services provided to children with neurological impairment whether of pre‐ or perinatal origin (i.e. cerebral palsy) or due to acquired brain injury sustained in later life.^1^ Despite the importance of neurorehabilitation treatments to the health care system, there is no consensus on how these largely non‐pharmacological interventions should be conceptualized or categorized, and their important ingredients are contested and difficult to define. This review focuses on the challenge of defining the active ingredients of *paediatric* neurorehabilitation and some emerging approaches for doing so. We mean ‘active ingredient’ in the same sense as a pharmaceutical treatment: the element(s) of the neurorehabilitation treatment that are known or hypothesized to be causally responsible for the intended clinical change.

In this review we will be using the terms ‘rehabilitation’ and ‘neurorehabilitation’ synonymously. We limit the scope of this discussion to interventions delivered to individual children with brain injury (or their carers) to enhance functioning. Thus, we exclude population‐level public health and policy interventions such as accessible public transport or special educational policy,^2^, ^3^ and also exclude broader usages of the term ‘rehabilitation’ referring to ‘physical (re)conditioning’ such as ‘cardiac rehabilitation’ and the concept of ‘early rehabilitation and mobilization’ in the intensive care literature.^4^ There will be significant overlap in this review with what has been called ‘habilitation’ (therapeutic work with children with neurological impairments present from birth, i.e. cerebral palsy), although that term is not widely used currently.

## THE PROBLEM

Children with neurological injury typically demonstrate functional change even without rehabilitation, as a result of ‘spontaneous’ recovery early after acquired brain injury and, over longer timescales, physiological development and maturation. In order to understand and ultimately improve the effectiveness of rehabilitation interventions, we need to be able to detect treatment effect signals (i.e. the ‘difference rehabilitation has made’) against the noise of patient and injury heterogeneity and change over time.^1^ This requires us to be able to identify the extent to which a child's recovery is better (or indeed worse) than expected (i.e. the residuals from outcome‐prediction models that omit the rehabilitation delivered)^5^ and relate this to rehabilitation treatment. Although imperfect and incomplete, we have at least preliminary approaches to characterizing the site(s) and severity of a child's injury,^6^, ^7^ and the injury‐independent physical, environmental, and social determinants of health.^2^, ^8^, ^9^ Similarly, we have increasing consensus on important constructs in health outcome^10^ and improving measures of functioning both globally and in subdomains, and evolving common data elements that allow us to measure these outcomes of the rehabilitation process.^11^

In contrast there is little consensus on how to define and measure the ‘input’: the content of rehabilitation treatment and its dose. Current pragmatic approaches to rehabilitation treatment definition such as time spent in a given type of facility (x days of inpatient/residential rehabilitation) or with a specific discipline (x hours of occupational therapy) are inadequate as they ‘black box’ the intervention.^12^ Even labels suggesting defined theoretical approaches (x sessions of Bobath therapy) have been increasingly recognized as inadequate: in the specific case of Bobath there is disagreement between practitioners as to what exactly this comprises, or, perhaps more importantly, what if anything would now be excluded by the term.^13^

‘Problem‐based’ treatment names such as ‘dressing practice’, ‘aphasia therapy’, or ‘executive function training’ are similarly inadequate as treatment content descriptors (one would not consider dieting and exercise programmes as equivalent forms of ‘obesity therapy’, or consider comparable the very different interventions that could be described as ‘stereotypy therapy’ for females with Rett syndrome).^14^ In the last decade or so there has been an important shift away from a bottom‐up focus on specific impairments (such as increased tone, movement synergies, or aphasia) to a top‐down ‘functional’ emphasis targeting activities with personal relevance and motivation (typically expressed as a focus on participation goals).^15^, ^16^ However, there is still a risk of defining the ‘why’ (the goal) without defining the ‘what’ or ‘how’ of rehabilitation content^17^ (see ‘Discussion’ below).

The ultimate effects of brain injury on the participation of a child and their family occur via many mediators (including but by no means limited to impaired motor and cognitive function, loss of family income and parental emotional health, and lack of equipment or adaptations) that may be countered by resilience factors.^18^ Rehabilitation is a complex package of interventions operating either in parallel or sequentially, targeting these various mediators via multiple ‘mechanisms of change’.^19^ Successfully targeting a mediator (e.g. improving reaching accuracy) is typically a necessary but not sufficient condition for improved participation. Thus for example, a trial discussed in more detail below that aimed to improve developmental outcomes in infants at high risk of developing cerebral palsy failed to meet its primary outcome although process evaluation confirmed that the targeted mediators (family involvement, stimulation of motor behaviour of demanding difficulty) were modulated.^20^

Without clear concepts of active ingredients and what might be expected to be changing as a result, it is difficult to align outcome measures with expected treatment effects (for this therapy should we be monitoring muscle strength, emotional health, walking speed, or executive function?) and to recognize where expectations of change are unrealistic (why should the motor function of this child with a severe disorder of consciousness improve if what we are primarily providing currently is emotional support for their mother?)^21^

The absence of frameworks for description of rehabilitation content complicates assessment of treatment fidelity (how do we define the criterion standard?), replication, and aggregation and meta‐analysis of studies. It also obscures the extent to which rehabilitation treatment content evolves over time, even when the therapy schedule remains constant (‘an hour of physiotherapy daily’). Such content changes may be driven at least partly by therapists' reassessments and changing expectations of recovery.^21^

For practitioner training this gap also complicates the definition of competent treatment in a given domain and the supervision of students and trainees to deliver treatments as intended. For clinical practice it leads to unclear communication: are different clinicians doing different things under the same name or the same thing under different names?^13^ Lack of linkage of ingredients to specific outcomes limits the ability to tailor to patients' individual differences. It also allows nurses, schoolteachers, and parents to think reductively that ‘rehabilitation is that thing therapists do, I can leave it to them’: to counter this we need to be able to specify rehabilitation content independent of who is delivering it.

There are overlaps between these issues and the broader, widely acknowledged challenge of adequately specifying complex interventions.^22^, ^23^, ^24^ However standard approaches to the latter (such as the TIDieR reporting guidelines)^24^ are too generic, lacking a rehabilitation treatment‐theory informed framework to ask cause–effect questions (‘which ingredients were responsible for the clinical change I observed?’)^25^

## THE DEVELOPMENTAL DIMENSION

Much of the impact of rehabilitation relies on various forms of learning, including explicit learning (acquisition of articulable knowledge) and implicit skill development through repeated practice (‘learning by doing’). In broad terms, the biology of relearning after injury is similar to that of first learning;^26^ however it is generally easier to relearn skills that had been established before injury than to learn abilities *de novo* in the presence of prior injury.^27^ The distinctions such as they are between habilitation and rehabilitation relate to this effect, together with the typical age at time of treatment, and the constraints this in turn places on possible treatment content: neither the very young nor the very impaired can cooperate with requests to practise specific activities (i.e. any rehabilitation activity with a volitional element); nor generally will explicit teaching of strategies (thought habits or mental representations) be feasible.

The International Classification of Functioning, Disability and Health emphasizes that the same impairments may result in very different functional outcomes depending on the facilitating or inhibiting effects of the physical and social environment.^2^, ^8^ This environmental impact is equally relevant for facilitating or inhibiting the performance of therapeutic rehabilitation activities. Thus, for younger or more impaired children, the focus is on (local) environmental optimization that supports participation in ways that in turn encourage attempted activity. For example, by providing an optimized environment (appropriate toys and levels of sensory stimulation with appropriate positioning, on a background of promotion of sleep and freedom from pain, etc.), play may be encouraged in a young child that in turn—even in the presence of neurological impairment—might for example promote repeated volitional reaching.

## EMERGING APPROACHES TO THE PROBLEM

Although there have been some attempts to define rehabilitation content in other contexts (e.g. adult spinal cord injury,^28^ adult stroke,^29^ and traumatic brain injury)^30^ we would argue that many still suffer from limitations discussed above, particularly ‘problem‐based treatment naming’. An important exception is the Behaviour Change Taxonomy,^31^ which provides a framework for parsing the core elements of more cognitively focused interventions, which of course play an important role in rehabilitation. The Behaviour Change Taxonomy has been used to analyse aphasia therapy content^32^ and is incorporated into the Rehabilitation Treatment Specification System (RTSS).

In selecting the examples below we have prioritized approaches that are ‘treatment theory based’ in that they define combinations of active ingredients and domains of function that those active ingredients are expected to affect based on knowledge of the treatment's mechanisms of action. They vary however in both their ‘granularity’ (zoomed in vs zoomed out) and comprehensiveness (coverage of the full range of potential rehabilitation treatments). The approaches we have personally been most involved with (the RTSS and Paediatric Rehabilitation IngredientS Measure [PRISM]) are intended to be comprehensive for paediatric neurorehabilitation (PRISM would need slight modification for adult neurorehabilitation), but differ greatly in their granularity. Others are intentionally more focused and less comprehensive in scope.

### The Rehabilitation Treatment Specification System

The RTSS was developed as a conceptual framework for consistent specification of all rehabilitation treatments. The initial intent was to develop a full rehabilitation treatment taxonomy^33^, ^34^ but it was recognized that treatments must be defined systematically before one can consider how to group them—that specification must precede development of a taxonomy. And the challenges of specification alone loomed so large that the project's efforts shifted, at least in the interim, to this task.

The RTSS is designed around treatment theories, statements of how active ingredients change a treatment target through a mechanism of action. Accordingly, the RTSS defines rehabilitation treatments with respect to their known or hypothesized active ingredients; and requires that both active ingredients and treatment targets must be observable and measurable, at least in principle, which requires that they be operationally defined. Treatment theories specify effects of treatment that in principle are universal (will be seen in all patients meeting specifiable criteria) and direct (do not involve intermediate effects).

Importantly, the RTSS is agnostic about any hoped‐for downstream effects of treatments which are, in turn, specified by enablement theories:^35^ views of whether and how changes in one target might affect ‘downstream’ areas of functioning in particular patients. Whereas a given antispasticity treatment might reduce passive stiffness of a joint (a treatment target) in two patients, it might facilitate skilled movement for one child but not the other (a distal treatment aim), if one child has a disorder of consciousness and the other does not.

As discussed above, most if not all rehabilitation treatments can be classified as ‘complex’ interventions, with subcomponents intended to modify inter‐related functions to achieve an overall effect. One might, for example, provide a treatment that includes strengthening exercises, reaching practice, and family education about the importance of regular performance of these exercises. Although the aggregate effect may be more spontaneous reaching behaviour, the overall treatment addresses multiple distinct targets of increased muscle strength, improved reaching accuracy, and enhanced caregiver knowledge and motivation. Each treatment target, along with the active ingredients addressing it, is considered a treatment component.

The RTSS posits that all rehabilitation treatment comprises components falling into one or more of three major treatment groups distinguished by their central mechanisms of action (thus forming the beginning of a treatment taxonomy):^36^ (1) Organ functions treatment components modify the functioning of organs or organ systems (upregulation, downregulation, etc.), or substitute for their function (as with prostheses), typically with the delivery of physical ingredients such as application of force, radiation, or chemical substances. (2) Skills and habits treatment components enhance the efficiency of mental and physical skills and/or make their performance more automatic. The central ingredient for these treatments is repeated performance (practice), often with various forms of guidance and feedback. (3) The representations treatment group includes treatments intended to modify articulatable knowledge, attitudes, beliefs, and motivations through the delivery or guided reprocessing of information. The RTSS also includes specific guidance for characterizing the important large number of rehabilitation treatments that are volitional in nature, in that they depend for their effects on effortful performance by the patient. For such treatments, one must specify not only the active ingredients involved in changing a specific target (e.g. contracting a set of muscles against resistance to increase strength), but also the active volition ingredients that ensure the performance of the necessary treatment activities (e.g. game‐like activity that encourages repeated muscle contraction).^37^ It is these instructional and motivational ingredients that are borrowed from the Behaviour Change Taxonomy^31^ and that are typically viewed as comprising elements addressing capability (optimizing the challenge gap between ability and goal), opportunity and motivation for the behaviour (the so‐called COM‐B model of the Behaviour Change Taxonomy).^38^

**FIGURE 1:**
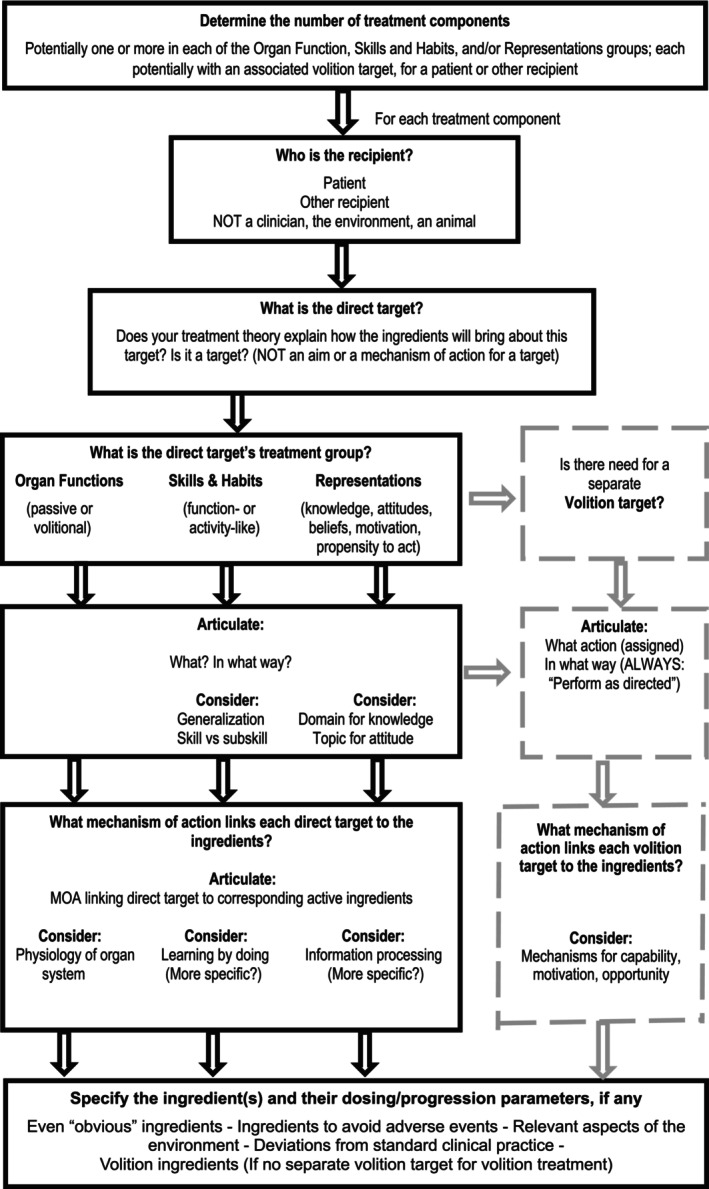
The process of specifying a rehabilitation treatment using the Rehabilitation Treatment Specification System. The treatment is divided into distinct treatment components, each with an operationally defined target and a set of corresponding ingredients. The mechanisms of action that link the selection of ingredients to their corresponding targets are at variable and evolving levels of precision, ranging from manipulation of specific physiologic processes to ‘learning by doing’.

Although changes in skills and mental representations clearly occur in the brain, RTSS views the targets as the actual changes in skills or representations, with any underlying neuroplastic brain changes viewed as mechanisms of action that help account for the behavioural changes. Only for certain entirely ‘passive’ (non‐volitional) treatments such as administration of a psychoactive medication or electromagnetic stimulation would the ‘brain’ be regarded as the treatment target and this would be an organ functions group intervention. With this in mind, the process of treatment specification using the RTSS involves dividing up the treatment into individual components each with a single target; identifying the treatment group for each treatment component; specifying each particular target as appropriate for its group; specifying the hypothesized active ingredients for each treatment component, including volition ingredients where relevant; and specifying dosages in theory‐relevant ways.^39^ The specification process is shown in flow‐chart form in Figure 1.

### The Paediatric Rehabiliation IngredientS Measure

PRISM, with an intended metaphor of splitting light into colours, uses a simple method to help clinicians estimate the proportions of a team's ‘total rehabilitation effort’ allocated across a menu of 11 possible broad rehabilitation content options listed in Table 1 (users are encouraged to limit to four or five the number they consider relevant in a given assessment).^40^ One goal of PRISM was to reflect the 24/7 efforts of the whole rehabilitation team particularly in residential/inpatient contexts, for example capturing the therapeutic actions outside ‘office hours’ of nursing and care staff and avoiding over‐emphasis on direct therapist contact time.

**TABLE 1:** The ingredient‐mediator matrix providing the origin of the menu of 11 possible classes of rehabilitation content in the PRISM system.

	Child activity and function	Family	Physical environment	Attitudinal environment
Supported practice and repetition (‘learning by doing’)	✓	✓	NA	NA
Education (support of explicit learning)	✓	✓	NA	✓^a^
Other management of body structure and function	✓	NA	NA	NA
Emotional health support	✓	✓	NA	NA
Adaptation	NA	✓^b^	✓^c^	✓^d^

For further information see Forsyth et al.^40^

Abbreviation: NA, not applicable; PRISM, Paediatric Rehabilitation IngredientS Measure.

^a^ explicit education or training of professionals external to the rehabilitation therapy team.

^b^ adaptation within family home.

^c^ adaptations outside family home (e.g. at school).

^d^ advocacy with (health, education etc.) professionals external to the rehabilitation therapy team.

PRISM's development was shaped by a more specific research agenda than that of the RTSS, namely the study of possible dose–response effects in paediatric rehabilitation. One rationale for PRISM's approach is that dose–response relationships are likely to vary across these different types of rehabilitation content: they are likely to be strongest for learning by doing (consider the analogy of practising a musical instrument where arguably one never stops learning) but for explicit learning (e.g. a parent learning how the special educational needs system works) one would expect saturation effects (once you know something, being repeatedly retold it is not useful). And for indirect ‘advocacy’ work (arguing with an insurer for funding of a period of inpatient rehabilitation) dose–response effects are relatively meaningless. Failure to acknowledge these distinctions may be one reason for the relatively weak evidence of dose–response relationships to date in adult rehabilitation studies.^41^

### Others

A notable early attempt to develop a treatment theory based classification of (re‐)habilitation content is the work of Blauw‐Hospers et al.^42^ This focused specifically on physical therapy interventions for very young infants at risk of developing cerebral palsy, a much narrower scope than either PRISM or RTSS. The observation schedule developed identified eight mutually exclusive elements of therapy content across neuromotor and family involvement and education elements. From this they were able to develop a theoretically‐informed therapy programme (‘COPing with and CAring for infants with special needs’, COPCA) and confirm its distinctiveness from ‘traditional’ approaches. Importantly the observation schedule was able to demonstrate to therapists' surprise that they were not always doing what they planned or thought they were doing. Their (arguably underpowered) randomized controlled intervention trial failed to meet its primary developmental endpoint (an improved Infant Motor Profile score); however, their tool provided the treatment content resolution to identify post hoc correlations between selected features of therapy content (amounts of parent coaching, challenging infants to initiate and continue behaviour, and stimulation of motor behaviour at the limit of the infant's capability) and the Infant Motor Profile score.^20^, ^43^ As with the PRISM work,^21^, ^44^ however, an important causal‐inference challenge remains: were the treatment content differences responsible for better motor function, or were therapists responding to observed better motor function in their treatment‐content choices?

Motor learning theories have probably been the most fruitful perspective in the wider paediatric (re‐)habilitation characterization literature. General motor learning insights emphasize characteristics of chosen practice tasks (the extent to which they are goal‐related, challenging, and motivating; and whether they are open or closed, discrete or continuous); characteristics of practice (e.g. blocked vs random, whole or part, length, duration, and frequency; graduation of difficulty; error‐based or errorless); and the nature of feedback.^45^, ^46^ The Motor Learning Strategies Rating Instrument^47^ provides a taxonomy of motor learning strategies, including verbalizations (e.g. nature of spoken encouragement, instruction, or feedback); therapist actions (e.g. demonstration, guidance); and nature of practice (e.g. blocked vs random sequencing, use of progression and repetition). These can be used to characterize the content of observed therapy sessions, and also usefully to attempt to ‘deconstruct’ described therapeutic approaches or philosophies to allow their core features to be compared and contrasted.^46^ The Motor Learning Strategies Rating Instrument has been used to characterize motor learning strategies in the context of neurorehabilitation after paediatric acquired brain injury.^48^

## POTENTIAL AND CHALLENGES FOR THE FUTURE

In principle the RTSS could address many of the problems alluded to above, by aligning treatment labels with the active ingredients required for achieving their results. In a research context, clear specification of the relevant treatment theories supports fidelity (through clarity about the treatment ingredients that must be delivered), clarity about appropriate study populations (they must be able to respond to the treatment's mechanism of action), and specification of a meaningful comparator treatment (by clearly identifying the ingredients that must be markedly reduced or absent in that comparator). It also informs selection of outcome measures aligned with the treatment target, and should facilitate aggregation of evidence and meta‐analyses by clarifying the ingredients that various treatments have in common. The rigour of the RTSS should also help distinguish treatment failures due to ineffective treatment ingredients from failure to engage in the treatment as directed (i.e. volition failure). At a broader level, the existence of distinct treatment classes invites the formulation of research questions that cut across multiple treatments such as whether there are general characteristics of efficient practice across different skills and habits treatments.

In principle the RTSS can also support clinical education by promoting explicit discussion of treatment theories and allowing for supervision of the delivery of appropriate ingredients. However, clinician feedback has been clear that whilst conceptually illuminating, the RTSS is complex to learn and apply in day‐to‐day clinical practice. Clinicians tend to ‘bundle’ interrelated actions and parsing them into distinct treatment components can be time‐consuming. The levels of detail required for research may not be required or feasible for routine clinical use and documentation.

The rigour of rehabilitation content specification also highlights the challenge of specifying rehabilitation dose and the inadequacy of metrics such as ‘number of sessions’ or ‘minutes’. Recommendations to specify treatments' Frequency (repetition rate), Intensity (‘strenuousness’), Time (session duration), and Type^49^ are a starting point; however different therapies have their own best ‘dose’ metrics and these are largely incommensurate. It would be very challenging, for example, to compare a dose of splinting, or constraint therapy, to a dose of active practice.^15^, ^50^ The RTSS specifies dosage in units relevant to the treatment theory at hand (e.g. units of force or energy intensity for an organ functions treatment, practice schedule, and repetitions for a skills and habits treatment, etc.). As discussed above, dose–response relationships will differ between intervention types and are probably meaningless for some. Even where dosage carries some quantitative notion, it may not necessarily be a linear relationship as implicitly assumed by frequency, intensity, time, and type approaches (is half as much for twice as long equivalent?):^51^ given the complex biology of the underlying processes of neuroplasticity^52^ this is unlikely to be the case.

RTSS and PRISM illustrate an important ‘granularity’ tension between precision in specification and feasibility. A researcher developing a single new treatment can afford to specify its ingredients in detail whereas a practising clinician seeing multiple patients a day is unlikely to have the time to think at that level of detail, much less document treatment accordingly. On the other hand, even a PRISM‐level consideration of whether a session is achieving active task practice by a child or education of a parent sharpens precision over a ‘one hour of occupational therapy’ approach and, as we have found, provides important insights^21^, ^40^ and promotes a whole‐team ‘synoptic’ perspective. Although PRISM and RTSS have very different levels of granularity, they have similar conceptual frameworks. In PRISM there is explicit specification of both child and family member as distinct potential recipients of treatment; in the RTSS the recipient must be defined on each occasion (in adult rehabilitation this is most often the client but may be a carer). More importantly, PRISM ‘bundles’ two classes of treatment that the RTSS requires practitioners to ‘unpack’. PRISM's ‘emotional support’ (whether for the child or family member) would be considered by the RTSS as combinations of information‐driven changes in knowledge and attitudes (representations), developing psychological ‘skills and habits’ through repeated performance and possibly even organ function interventions (such as prescription of an antidepressant): considering emotional interventions as differing in their targets, but not fundamentally in their active ingredients, from more physically and cognitively focused interventions. Elements of the RTSS representations category apply across all of the PRISM intervention categories where voluntary effort is required. Perhaps the RTSS's approach to interventions involving advocacy or environmental modification, which appear in the adaptation category in PRISM, is the most distinctive. RTSS's strict focus on treatment theory and mechanism, rather than enablement theory, is seen in its position that the effects of adaptations treatments too must ultimately converge exclusively on representations, skills and habits, and/or organ function treatments. For example adaptations may improve capability for tasks with volitional elements (by reducing the ‘challenge gap’ between ability and goal), together with possible skills‐acquisition elements (e.g. for an electric wheelchair), or the explicit instruction needed to benefit from them. Just how to ‘decompose’ the effects of a powerful intervention such as an adaptation is not rigidly specified: but we believe the challenge set by RTSS to do so leads to important discussion and precisely the careful analyses of treatment theory hypotheses we are calling for.

At present the RTSS offers a framework for parsing rehabilitation content but no ‘controlled vocabulary’ for describing specific targets or ingredients. Consequently, there is no easy way to distinguish between minor semantic differences describing essentially the same treatment from more substantive differences in targets or ingredients. There may be opportunities here for synergy between some of the approaches described above: for example the Motor Learning Strategies Rating Instrument identifies some important rehabilitation ingredients that would fall in both RTSS's skills and habits and representations classes. Consensus‐based menus of mutually exclusive operationally defined targets and ingredients will be needed, particularly for implementation in electronic medical record systems.

## DISCUSSION

Rehabilitation research is held back by the lack of a coherent and practicable system for defining existing and emerging treatments. As one of us (JW) has emphasized,^53^ this lack has driven clinicians to describe good rehabilitation at the process level (e.g. the occurrence of multidisciplinary team meetings or levels of clinician training and experience),^54^ but ultimately if units meeting these criteria are achieving better outcomes this must be because of resultant changes in the content of rehabilitation therapy as experienced by the injured child and their family: the multidisciplinary team discussions and/or clinician experience must lead to the implementation of superior therapy programmes, the content of which remain unexamined. Several of the recommendations in recently published practice guidelines for early interventions in cerebral palsy^16^ are open to similar criticism such as advice that therapy should include ‘client‐chosen goals, whole‐task practice within real‐life settings, support to empower families, and a team approach’. Again, precisely why and how such recommendations might lead to superior outcomes remains undefined and the subject of implicit assumption (e.g. perhaps parental empowerment might be increasing practice opportunity and thus ‘dose’; or that client‐chosen goals improve motivation). Similar criticisms can be made of the literature on early intervention for autism.^55^ Any theoretical underpinnings of a treatment choice (e.g. neurodevelopmental theories) can only be important insofar as they observably alter rehabilitation content: although rehabilitation treatment can be categorized in many different ways, we believe that only a system focused on known or hypothesized active ingredients of treatment (i.e. a mechanism‐based system) can accelerate research and promote evidence‐based practice. In a recent example from adult neurorehabilitation research, the RTSS allowed a useful meta‐analysis of treatments for presbyphonia (age‐related loss of speech volume) by distinguishing treatments with motor learning elements from muscle strengthening approaches.^56^

These considerations highlight the current lack of precision in many treatment theories. Whilst we can hopefully assume a clinician always has some rationale for selecting a given treatment for a given patient on a given day, this rationale may be a long way from the level of theoretical precision required by the RTSS. Is the target simply improvement in the activity that the child is engaging in? Or is the treatment benefit expected to generalize to related activities and, if so, how distantly related? The demand to make treatment hypotheses explicit has important conceptual value, by pointing out gaps in knowledge as well as formal similarities across a range of individual treatments. But at the practical level of being able to complete a written specification of a given treatment, vague theories nevertheless present an obstacle. Efforts are underway to address some of these obstacles for the RTSS and promote its greater adoption in research and practice, though most of the efforts at implementation have been in adult rehabilitation. A manual that guides treatment specification has been published and is currently being updated and revised to make its contents more accessible^39^ and RTSS principles^57^ are being incorporated into a set of trial‐reporting guidelines to be published by Cochrane Rehabilitation.^58^ The RTSS has been used successfully to examine shared and unique ingredients across a set of complex treatments in (adult) speech and language therapy.^59^ Small‐scale projects are under way exploring the development of operationally defined menus of treatment targets and ingredients that can further enhance multidisciplinary communication and provide the ontology for electronic medical record systems that should help extend the concept of a ‘learning health care system’ to rehabilitation. An example of RTSS parsing of a typical paediatric neurorehabilitation scenario is available in Appendix S1.

PRISM works at a much coarser level of granularity than RTSS but its simplicity supports quantitative analysis and data visualization (the application of PRISM is demonstrated at https://www.youtube.com/watch?v=‐GSjSiM_apE). For example it has allowed confirmation of important relationships between therapists' often implicit expectations of recovery and their therapy content decisions; and between those content decisions and ongoing recovery.^21^, ^44^ It has also allowed visualization and quantification of the often striking extent to which rehabilitation content changes over time at least during inpatient rehabilitation admissions, presumably at least partly in response to observed recovery,^44^ something that has also been noted in other contexts.^60^ Such evolution in treatment content is largely invisible to metrics of rehabilitation input such as hours spent with therapists from given disciplines. PRISM can quantify ‘magnitude of change in treatment content’ which may be a metric worthy of further study, and an important area of future research will be examination of reasons for such treatment content change decisions by therapists.

PRISM particularly highlights how rehabilitation treatment content may be shaped by therapists' prior expectations of functional change: expectations that may be influenced by the observed response to treatment so far. This highlights the challenge of randomized controlled trials in rehabilitation research: rigidly ‘protocolized’ therapy that cannot be adjusted in response to clinical reassessment risks being seen as a pale shadow of ‘good’ therapy. Conversely there is a major causal inference challenge in demonstrating dose–response effects for rehabilitation that is allowed to evolve in light of observed recovery^21^, ^44^, ^50^ and we are currently working on this.

A system of treatment definition is ultimately a communication system, and it cannot achieve its full impact unless its language is adopted by researchers studying treatments and clinicians applying them. As discussed, emerging systems for treatment definition have overlapping but distinct conceptual features as well as differing levels of granularity. Many challenges remain in refining and implementing these emerging systems to the point that they support rigorous rehabilitation research and a learning rehabilitation system.

## Supporting information


**Appendix S1:** RTSS specification example

## Data Availability

Data sharing not applicable ‐ no new data generated, or the article describes entirely theoretical research.
