# Supplementary material for: Defining paediatric neurorehabilitation: You cannot improve what you cannot characterize
Source: Dev Med Child Neurol. 2024 Apr 26;66(9):1123–32. doi: 10.1111/dmcn.15919 (PMC11579808; doi:10.1111/dmcn.15919)
Supplement: Supplementary file 1 — Appendix S1: RTSS specification example [file DMCN-66-1123-s001.docx]

**Supplemental Data: RTSS Specification Example**

This is a worked example of specifying neurorehabiliation interventions using the RTSS framework. We begin with a clinical vignette, followed by suggested RTSS specifications, from the perspective of the treating therapist(s). This means that the RTSS specification is done with knowledge of the underlying rationale and treatment theories. An external observer may not be able to determine the targets and active ingredients by observation alone, particularly within a single session, just as an observer of a medication treatment may not be able to identify the active ingredient or the target being treated.

VIGNETTE

Emily is a twelve year old previously right-handed girl who presented with an acute onset right hemiplegia four weeks ago due to rupture of a previously unsuspected arterio-venous malformation. The hemiplegia affects her right arm more than her right leg. She can open the fingers of her right hand but has limited manipulation skills. She can walk short distances on smooth indoor surfaces but finds this fatiguing, and a plantar flexion contracture of the ankle and hypertonia of the ankle and knee extensors sometimes exacerbate her movement difficulties. She has a significant aphasia. Therapeutic intervention is directed toward improving upper and lower extremity mobility and communication.

**Constraint-induced movement therapy (CIMT) to enhance functional use of right hand and arm**

Group = O (Organ), S&H (Skills and Habits), R (Representations), R(V) Representations (Volition Target)

| **TARGETS** | | | | **INGREDIENTS** | | **MECHANISM OF ACTION** | |
| --- | --- | --- | --- | --- | --- | --- | --- |
| **What/In What Way** | | **Group** | | **(Dosing Parameters/Progression, when applicable)** | |  | |
| Increased functional use of paretic hand and arm | | S&H | | - Set of arm activities selected by Emily with agreed hierarchy of difficulty (V) - Demonstration and verbal cues of performance of each task (V) - Mitt placed on intact hand - Opportunities to repetitively practice right hand and arm activities (Timed practice of 2 tasks, 30 sec duration, x 10 reps with rests as needed) - Positive verbal reinforcement used to shape temporal and spatial accuracy - When Emily can perform 10 repetitions while maintaining good form, introduce the next most challenging task | | Learning by doing (expansion of cortical map representing the affected limb?) | |
| Completion of daily upper extremity activities at home as agreed | | R(V) | | - Verbal explanation of evidence supporting CIMT (initially and as needed) - Education about importance of the “transfer package” to facilitate carryover of treatment activities at home (initially and as needed) - Logbook and verbal instructions for recording performance of daily exercises - Negotiation with Emily and her parent(s) of a set of 2-3 desired and optimally challenging arm activities to be performed at home during 45-60 min practice sessions - Review of the logbook and discussion with Emily and her parent(s) about challenges to adherence and verbal reinforcement of successful performance (each session) | | Information processing | |
| Increased parental confidence in facilitating CIMT adherence at home | | R | | - Discussion of the importance of adherence (as above) - Discussion of times/settings when constraint may be difficult and how to revise the activities or schedule - Discussion of taking breaks, interspersing with episodes of non-manual activities, offering positive reinforcement for mastery - Developing reward system for Emily’s completion of daily logbook and UE activities | | Information processing | |

Notes: The primary Skills and Habits treatment component involves practice of manual activities with the paretic hand while the other hand is restrained, and a “transfer package” of negotiated carry-over activities to be practiced at home. The clinician considers a more specific treatment theory than simply “learning by doing”, based on animal and human research showing plastic expansion of cortical movement areas and greater activation of the injured hemisphere in response to practice. There are ”volition ingredients” (V) in this treatment component, selected to enhance Emily’s engagement with the practice tasks. Dosage of the practice is represented both by the number of practice trials and by a rough progression algorithm that introduces more challenging tasks as Emily’s performance improves. Because the home practice depends on the understanding and motivation of Emily and her parent(s), generated within the treatment session, there is a separate volition target (“R(V)”) of performing the necessary home practice, with corresponding informational and motivational ingredients. There is also a separate Representations target directed at the parent(s), to equip them with information that may be helpful in supporting the child’s practice.

**Serial casting to improve passive dorsiflexion range at the right ankle**

Group = O (Organ), S&H (Skills and Habits), R (Representations), R(V) Representations (Volition Target)

| **TARGETS** | | **INGREDIENTS** | **MECHANISMS OF ACTION** |
| --- | --- | --- | --- |
| **What/In What Way** | **Group** | **(Dosing Parameters/Progression, when applicable)** |  |
| Increased passive right ankle dorsiflexion range of motion | O | - Fiberglass - Cast applied in maximum tolerated ankle dorsiflexion with knee extended - Cast changed every 5 days until less than 5 degrees are gained on 2 successive changes; then remove and create resting bivalve cast - Involve Emily in choosing colour of and design on cast (V) - Cotton padding around areas of bony prominence while casting (S) | Prolonged stretch of soft tissues causes elastic elongation and increased collagen synthesis |
| Increased parental understanding of how to ensure cast safety | R | - Discussion of limb positions to minimize minor swelling; watching for color changes, persistent complaints of pain - Demonstration of where and how to check pulse | Information processing |

Notes: The Organ Functions treatment component involves *progression*, seen with many Organ Functions treatments, in which the demands of the intervention (i.e., angle of the cast) are adjusted as the target (i.e., passive dorsiflexion) improves. The therapist attributes the effects of casting to a combination of elastic stretch and new tissue synthesis. Involving Emily in choosing cast materials and placing padding over bony prominences, unlike the casting itself, have no mechanism of action directed toward range of motion. The former is intended to enhance Emily’s cooperation with the treatment and the latter to prevent skin breakdown from excessive pressure. However, by clerical convention, the RTSS allows these “volition ingredients” (V) and “safety ingredients” (S) to be included with the ingredients that are responsible for the treatment effect (prolonged stretch). An additional treatment component involves educating the parent(s) on safety monitoring.

**Self-management of Emily’s spasticity**

Group = O (Organ), S&H (Skills and Habits), R (Representations), R(V) Representations (Volition Target)

| **TARGETS** | | **INGREDIENTS** | **MECHANISMS OF ACTION** |
| --- | --- | --- | --- |
| **What/In What Way** | **Group** | **(Dosing Parameters/Progression, when applicable)** |  |
| Increased understanding of factors affecting limb spasticity | R | - Written pamphlet about spasticity, including discussion of positioning, stretching, concurrent illness and noxious stimuli - Discussion with Emily about her impressions of factors affecting tone in her limbs | Information processing |
| Increased understanding of the roles of oral medications and self-stretching (i.e. using unaffected limb to move affected limb passively through range of motion) in managing spasticity | R | - Discussion and Q&A of use of self-stretching at start of day, prior to key activities, at times of exacerbation - Discussion and Q&A of longer-term fluctuations, spasticity during the night, in relation to medication use | Information processing |
| Increased accuracy in performing self-stretching activities | S & H | - Clinician modeling of self-stretching activities (V) - Direction to stretch until it is uncomfortable but not painful (V) - Opportunities for Emily to practice self-stretching with verbal and tactile correction as needed (until able to perform with correct form) - Verbal encouragement and reinforcement (each trial) | Learning by doing |
| Taking medication at home as directed | R(V) | - Discussion of the correct dose of baclofen and demonstration how to lay it out in the pillbox - Discussion to plan most convenient placement of pillbox and times for administration - Medication pillbox | Information processing |
| Regular parental monitoring of medication self-administration | R(V) | - Review of the pillbox strategy with the parent(s), including when Emily should fill it and when to check it - Check sheet for documentation - Request to check the pillbox twice a day for the coming week, note any missed pills, and ask Emily to take them when noted | Information processing |
| Increased confidence in the ability to self-manage spasticity | R | - Review chart on reliability of medication self-administration by Emily, focusing on reinforcing successes and problem-solving missed doses - Discuss with Emily when she performed self-stretching and how helpful it was; discuss barriers and facilitators to self-stretching - Discuss with Emily current level of confidence in self-management of spasticity and plan goals for any remaining barriers. | Information processing |

Notes: The clinician has two educational targets for Emily, focusing on factors that might affect her spasticity and how to use self-stretching and medication to control her spasticity. Implicit in the “discussion” and “Q&A” is the notion that the discussion will continue until a criterion of understanding has been reached. A separate Skills and Habits target involves Emily learning to stretch her own right upper and lower limbs to reduce spasticity. Volition ingredients (V) are included to ensure that Emily knows how to perform the activity correctly. Here, similarly, the dose is until a performance criterion is reached. All 3 of these components are delivered in a treatment session such that the clinician can continue providing information or practice until satisfactory performance is achieved. In contrast, the next 2 treatment components pertain to behaviors that are to take place outside of the treatment session by the child and the parent. As above, these have volition targets reflecting the necessary home behaviors. Finally, the clinician believes that explicitly reviewing and discussing Emily’s performance at the start of each session will enhance her reliability and confidence in her self-management abilities.

**Naming practice to improve word-finding**

Group = O (Organ), S&H (Skills and Habits), R (Representations), R(V) Representations (Volition Target)

| **TARGETS** | | **INGREDIENTS** | **MECHANISMS OF ACTION** |
| --- | --- | --- | --- |
| **What/In What Way** | **Group** | **(Dosing Parameters/Progression, when applicable)** |  |
| Increased accuracy in naming objects of personal importance | S & H | - Opportunities to practice naming the pictures; if Emily fails, her parent supplies the name and asks Emily to repeat it (practiced 4 times/day, beginning with the 4 cycles close together and attempting to space them out more as accuracy improves during the week) | Learning by doing (strengthened connections between semantic features and word form, boosted by effortful retrieval) |
| Home practice by Emily of naming exercises as directed | R(V) | - Discussion with Emily and her mother about the need for daily practice of specific words of importance - Discussion of the importance of “effortful retrieval” (i.e., to space out the practice so that it is challenging but Emily usually succeeds) - Discussion of most convenient times and places for home practice - Involvement of Emily and her mother in selecting 10 named objects each week that are 1) difficult for Emily to retrieve; and 2) important in Emily’s daily communication - Involvement of Emily and her parent(s) in copying pictures of those named objects from the internet to the family’s computer - Instruction and modeling of the task, playing the role of the parent coach - Review of progress on last week’s words and reinforcement for both practice and improvement - Problem solving of any obstacles to home practice | Information processing |
| Home coaching of Emily’s naming exercises as directed | R(V) | - Discussion with Emily and her parent about the need for daily practice of specific words of importance - Discussion of the importance of “effortful retrieval” (i.e., to space out the practice so that it is challenging but Emily usually succeeds) - Discussion of most convenient times and places for home practice - Review of progress on last week’s words and reinforcement for both practice and improvement - Problem solving of any obstacles to home practice | Information processing |

Notes: The primary treatment component involves practicing naming common objects from pictures. The clinician’s theory is that repeated naming with correction and repetition when errors are made, will enhance the ability to retrieve the practiced words, and that the benefit of this practice is greatest when retrieval is effortful but correct. Note that the clinician’s treatment theory does not predict improvement in non-practiced words. The clinician specifies a dose in terms of the number of repetitions and recommends a rough progression by which the practice trials are gradually more and more spaced out, placing greater and greater demands on memory. This practice will take place at home but, as above, there are volition targets for both Emily and her parent, reflecting the performance of that home practice and home coaching as directed. The clinician involves Emily and her parent in choosing the words and pictures because she believes that this will enhance motivation to continue to practice. Although the information and discussion will occur jointly with the parent and child, with similar ingredients, they are expressed as separate targets since, in principle, they may need slightly different information and either or both of them could be the source of a future adherence problem.
